# Abnormal expression of CDK11^p58^ in prostate cancer

**DOI:** 10.1186/1475-2867-14-2

**Published:** 2014-01-08

**Authors:** Yayun Chi, Lisha Wang, Xiuying Xiao, Ping Wei, Yiqin Wang, Xiaoyan Zhou

**Affiliations:** 1 Cancer Institute, Fudan University Shanghai Cancer Center, Shanghai 200032, China; 2 Department of Pathology, Fudan University Shanghai Cancer Center, Shanghai 200032, China; 3 Department of Oncology, Shanghai Medical College, Fudan University, Shanghai 200032, China

**Keywords:** CDK11^p58^, Metastasis, Prostate cancer, Androgen receptor

## Abstract

**Background:**

CDK11^p58^ is one of the large families of p34cdc2-related kinases whose functions are linked with cell cycle progression, tumorigenesis and apoptotic signaling. Our previous investigation demonstrated that CDK11^p58^ repressed androgen receptor (AR) transcriptional activity and was involved in the negative regulation of AR function.

**Methods:**

CDK11^p58^ expression was examined in the prostate cancer tissues and adjacent tissues by IHC and qRT-PCR. Cell apoptosis was detected by flow cytometry. The metastasis of cancer cells was evaluated by the Transwell Assay. Finally we further investigated the underlying molecular mechanisms by examining expression levels of relevant proteins using western blot analysis.

**Results:**

We found that both RNA and protein expression of CDK11^p58^ were low in prostate cancer tissues compared with its adjacent noncancerous tissues. CDK11^p58^ promoted the prostate cancer cell apoptosis and inhibited its metastasis in a kinase dependent way. And finally CDK11^p58^ could inhibit the metastasis of AR positive prostate cancer cells through inhibition of integrin β3 and MMP2.

**Conclusions:**

These data indicate that CDK11^p58^ is an anti-metastasis gene product in prostate cancer.

## Introduction

CDK11, which is encoded by two highly homologous p34cdc2-related genes, Cdc2L1 and Cdc2L2 [1,2], and is known as PITSLRE protein kinase due to the conserved PITSLRE motif within the protein kinase domain [3]. CDK11^p58^ is one isoform of CDK11 and is closely related to cell cycle arrest and apoptosis in a kinase-dependent manner [2,4]. Previous studies revealed that CDK11^p58^ promotes centrosome maturation and bipolar spindle formation [5,6]. Our study has shown that cyclin D3 is vital for the kinase activity of CDK11^p58^. Cyclin D3 and CDK11^p58^ were involved in the regulation of AR-mediated transactivation. Cyclin D3/CDK11^p58^ holoenzyme kinase complex repress AR function through phosphorylating AR at Ser-308 [7]. Also, we found that CDK11^p58^ was autophosphorylated at Thr-370. Thr-370 is responsible for the autophosphorylation, dimerization, and kinase activity of CDK11^p58^[4].

Dysregulated AR signaling is implicated in several types of tumor, including carcinomas of the prostate, breast, liver and bladder [8]. Abnormal AR expression in prostate cancer are correlated with metastasis and aggressiveness [9]. Tumor metastasis is the main cause of lethality of prostate cancer, because conventional therapies like surgery and hormone treatment rarely work at this stage [9]. Tumor cell migration, invasion and adhesion are necessary processes for metastasis [10]. Metastasis is a consequence of many biological events, during which cancer stem cells are shifted into a malignant state [9,11]. Previous reports showed that an activated Wnt/β-catenin pathway and AR expression in prostate cancer are correlated with metastasis and aggressiveness. In addition, the expression of MMP-7 protein, a target of the Wnt/β-catenin pathway, is associated with PSA. AR is involved in the metastasis and invasiveness of prostate cancer. The overexpression of AR promotes the migration and invasion of prostate cancer cells [12]. As CDK11^p58^ was involved in the negative regulation of AR, we speculated that CDK11^p58^ might be involved in the regulation of prostate cancer metastasis.

In the current study, we further examined the expression in the prostate cancer tissues and investigated the biological functions of CDK11^p58^ in prostate cancers. Our study indicated that expression of CDK11^p58^ was decreased in the prostate cancer and CDK11^p58^ was involved in the negative regulation of prostate cancers.

## Results

### Expression of CDK11 in normal prostate tissues and prostate cancer tissues

**Figure 1:**
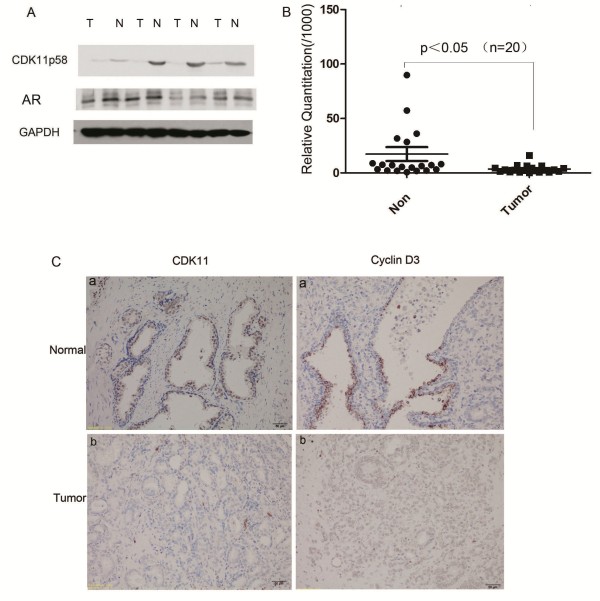
**Expression of CDK11 in normal prostate tissues and prostate cancer tissues. (A)** Prostate cancer tissues were lysed and subject to immunoblotting analysis as indicated. Protein levels were normalized to GAPDH. **(B)** 20 pairs of prostate cancer tissues and normal tissues were subject to qRT-PCR as described above. The relative mRNA level was showed above. P < 0.05. **(C)** CDK11 and Cyclin D3 immunostaining was performed in normal postate tissues **(a)** and its paired cancer tissues **(b)**; All photomicrographs were amplified 400×.

CDK11^p58^ is located on human chromosome 1p36.33, a region frequently mutated in various cancers. To investigate the potential role of CDK11^p58^ in prostate carcinogenesis, we examined the expression of CDK11^p58^ protein in prostate cancer tissues and adjacent non-cancerous tissues. By western blot assay, we found that CDK11^p58^ was higher in normal prostate tissues than in cancer tissues (Figure 1A). The qRT-PCR assay showed that the mRNA level of CDK11^p58^ was also higher in the normal tissues than in the cancer tissues (*P* < 0.05, Figure 1B). Immunohistochemical (IHC) staining of CDK11^p58^ protein was performed in 20 paired tumor/non-tumor clinical tissue samples. This analysis revealed that CDK11^p58^ was clearly expressed in nucleus. The expression of CDK11^p58^ was decreased in prostate cancer tissues compared with paired, normal prostate epithelium, with decreased staining intensity and a lower proportion of positively stained cells in prostate cancer tissue (6/20) versus normal tissue (14/20) (Figure 1C). A similar trend in the expression of Cyclin D3, a partner protein of CDK11^p58^, was also observed. These data indicate that CDK11^p58^ is down-regulated in prostate cancer.

### CDK11^p58^ inhibited the proliferation and promoted the apoptosis of LNCap cells

**Figure 2:**
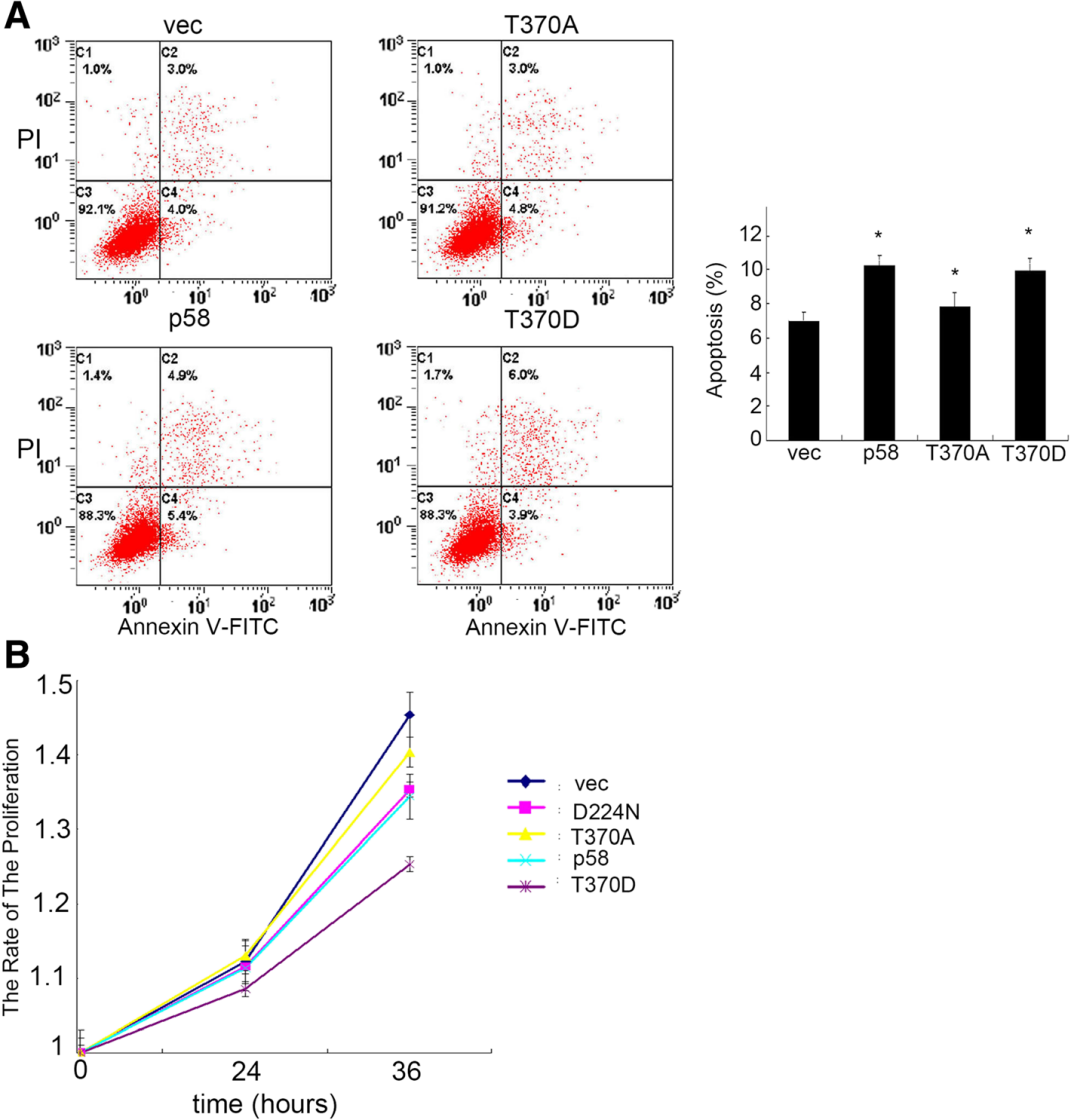
**CDK11**^**p58 **^**inhibited the proliferation and promoted the apoptosis of LNCap cells. (A)** LNCap cells were tranfected with indicated plasmids. 48 hrs later, the PI-Annexin V apoptosis assay was performed as described above. * *P* < 0.05 versus vector controls (n = 3 experiments). **(B)** LNCap cells were tranfected with indicated plasmids. 48 hrs later, the CCK-8 assay was performed as described above. *P* < 0.05 versus vector controls (n = 3 experiments).

CDK11^p58^ is one of the large family of p34cdc2-related kinases whose functions are linked with cell cycle progression, tumorigenesis, and apoptotic signaling. So we first examined the biological functions in prostate cancers. CCK-8 assay showed that CDK11^p58^ could inhibited the proliferation of LNCap cells. To test whether the inhibition was CDK11^p58^ kinase dependent, we used CDK11^p58^ mutants previously constructed in our laboratory (the kinase-dead mutant T370A, D224N and the kinase-active mutant T370D). Compared with the control, T370A, D224N, wild type CDK11^p58^ inhibited the proliferation of prostate cancer cells. The kinase constantly activitied mutant T370D exhibited even more inhibition effect than others (*P* < 0.05, Figure 2B). Also the apoptosis effect was tested. Wild type CDK11^p58^ and T370D could promote the apoptosis of LNCap cells but T370A failed to promote the cancer cell apoptosis (*P* < 0.05, Figure 2A). These data suggested that CDK11^p58^ promoted the apoptosis of prostate cancer cells and the effect was kinase dependent.

### CDK11^p58^ inhibited the metastasis of LNCap cells

**Figure 3:**
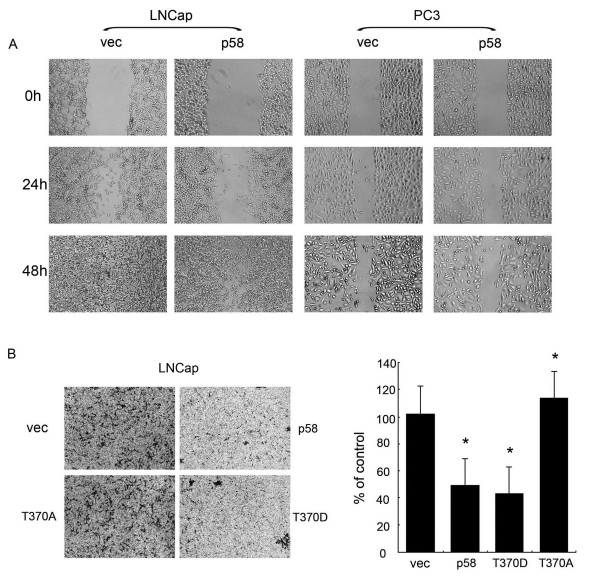
**CDK11**^**p58 **^**inhibited the metastasis of LNCap cells. (A)** LNCap cells and PC3 cells were transfected with pcDNA3.0 or CDK11^p58^. Six hrs after transfection, a wound was stimulated by a pipette tip. Migration was monitored for 12 hrs. The distance was measured by phase-contrast microscopy. The data below were shown as mean ± SEM of the distances in n > 5 separate experiments areas. **(B)** LNCap cells were transfected with CDK11^p58^ and its mutants. Six hrs after transfection, transwell assay was carried on as described above. Crystal violet dye staining of LNCap cells that invased in the transwell assays was shown. The bottom data were shown as mean ± SEM of the number of cells invasion in n > 5 separate areas. * *P* < 0.05 versus vector controls.

CDK11^p58^, a G2/M specific protein kinase, has been shown to be associated with apoptosis in many cell lines. However its role in cancer invasion and metastasis remains unclear. We investigated the role of CDK11^p58^ in the migration and invasion of prostate cancer. Scar assay showed that CDK11^p58^ could inihibit the migration of AR + LNCap cells, however it failed to inhibit the migration of AR- PC3 cells (Figure 3A). This suggested that CDK11^p58^ might inhibit the cancer cell migration through AR signaling. Then the transwell assay was carried on. Invasion of LNCap cells transfected with wild type CDK11^p58^ and the T370D constitutively active kinase mutant was significantly lower, compared with control cells (*P* < 0.05; Figure 3B). In contrast, the T370A kinase dead mutant failed to suppress metastasis of LNCap cells. These data suggest that CDK11^p58^ inhibits prostate cancer metastasis in a kinase-dependent manner.

### Interaction between CDK11^p58^ and AR

**Figure 4:**
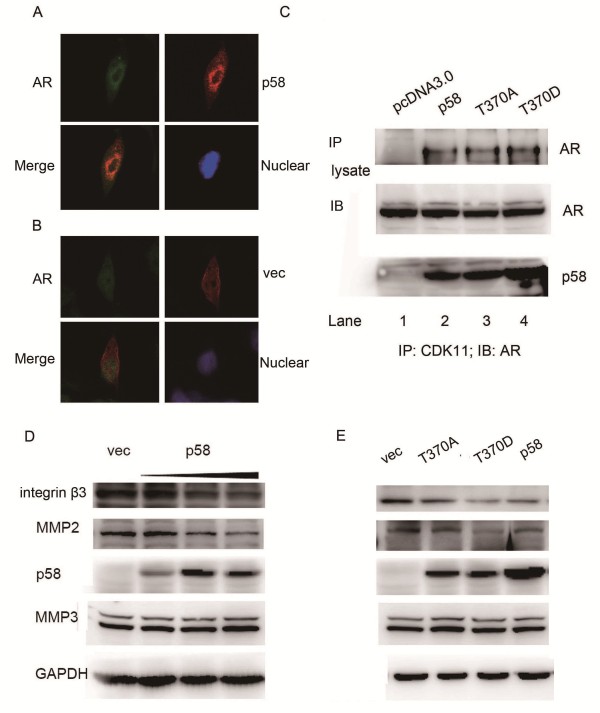
**CDK11**^**p58 **^**inhibited the expression of integrin β3 and mmp2. (A, B)** LNCap cells were transfected with CDK11^p58^. After 48 hrs, cells were subjected to immunoflurorescent staining assay. Cells were fixed and reacted with a mouse monoclonal anti-AR antibody and a rabbit polyclonal anti-CDK11 antibody. The secondary antibodies were anti-rabbit IgG-conjugated to fluorescein isothiocyanate and anti-mouse IgG-conjugated to rhodamine red. The images were captured with a Leica confocal microscope and software provided by Leica. **(C)** LNCap cells were transfected with CDK11^p58^ and its mutants. 48 hrs later, cells were lysed and subjected to immunoprecipitation with an anti-CDK11 antibody, followed by Western-blot analysis with an anti-AR antibody in the top panel. The bottom panels showed the expression levels of the AR and CDK11^p58^ from the prostate cancer tissue lysates. **(D)** LNCap cells were transfected with pcDNA3 and CDK11^p58^ with increased doses. After 48 h, cells were harvested and lysates subjected to immunoblotting analysis as indicated. Protein levels were normalized to GAPDH. **(E)** LNCap cells were transfected with wild type CDK11^p58^ or its mutants. After 48 h, cells were harvested and lysates subjected to immunoblotting analysis as indicated. Protein levels were normalized to GAPDH.

Previously, we showed that CDK11^p58^ interacts with AR in the postate cancer cell lines. In the present experiment, after pcDNA3.0-HA-CDK11^p58^ or pcDNA3.0 vector control were transfected in LNCap cells, we carried on the IF assay. As shown in Figure 4A, AR (green) and CDK11^p58^ (red) were localized mainly in nucleus. CDK11^p58^ was co-localized in the nucleus with AR (yellow). However, the control vector pcDNA3.0 (red) was not localized in nucleus and showed no colocalize with AR (Figure 4B). To further investigate whether the interaction between CDK11^p58^ and its mutants, we next performed Co-IP assays. The data showed that AR interacted with CDK11^p58^, T370D and even T370A (Figure 4C). However, in our previous study, the other kinase dead mutant D224N failed to interact with AR. These experiments demonstrated that CDK11^p58^ was capable of interacting with AR not necessarily dependent of its kinase activity. The exact mechanism needs further investigations.

### CDK11^p58^ inhibited the expression of integrin β3 and mmp2

We further examined the metastasis related protein involved in the metastasis signaling. Western blot analysis revealed that overexpression of CDK11^p58^ attenuated integrin β3 and MMP2 expression in a dose dependent mannar but not MMP3 (Figure 4D). Also the expression inhibition was kinase dependent (Figure 4E). These data indicated that CDK11^p58^ may repress integrin β3 and MMP2 to inhibit the metastasis of prostate cancer.

## Discussion

The incidence of prostate cancer (PCa), one of the most common cancers in elderly men, is increasing annually in the world. CDK11^p58^ is a mitotic protein kinase, which has been shown to be required for different mitotic events such as centrosome maturation, chromatid cohesion and cytokinesis [1-3]. Our previous study found that CDK11^p58^ repressed AR transcriptional activity and AR was phosphorylated at Ser-308 by cyclin D3/CDK11^p58^[4]. Furthermore, androgen-dependent proliferation of PCa cells was inhibited by cyclin D3/CDK11^p58^ through AR repression. D3/ CDK11^p58^ signaling is involved in the negative regulation of AR function. In this study, we demonstrated for the first time, that CDK11^p58^ expression is involved in the negative regulation of prostate cancer invasion in a kinase dependent manner.

Androgens and AR are indispensable for the development, regulation, and maintenance of male phenotype and reproductive physiology [5,6]. The AR signaling pathways play critical roles in the development and progression of PCa, a leading cause of cancer death second to lung cancer in men [7]. Metastasis is a consequence of many biological events, during which cancer stem cells are shifted into a malignant state. Dysregulated AR signaling is implicated in several types of tumor, including carcinomas of the prostate, breast, liver and bladder. Previous studies have showed that AR is involved in the metastasis and invasiveness of prostate cancer cells. The overexpression of AR promotes the migration and invasion of BFTC 909 cells. Inhibition of AR could inhibit AR-enhanced cell migration and invasion. Another study shows that an activated Wnt/β-catenin pathway and AR expression in PCa are correlated with metastasis and aggressiveness. Also AR mRNA expression shows significantly higher in prostate cancer when compared to benign prostatic tissue [8]. One report revealed the important roles of endothelial cells within the prostate cancer microenvironment to promote the prostate cancer metastasis and provide new potential targets of IL-6-- > AR-- > TGFbeta-- > MMP-9 signals to battle the prostate cancer metastasis [9]. Activated AR can downregulate E-cadherin expression to promote the activation of epithelial-mesenchymal transition and tumor metastasis [10].

As CDK11^p58^ could inhibit the transcription activity of AR, we speculated CDK11^p58^ could inhibit the metastasis of prostate cancer through AR signaling. First, we examined the expression of CDK11^p58^ in prostate cancer tissues to find that the expression of CDK11^p58^ was decreased in prostate cancer tissues compared with paired, normal epithelium, with decreased staining intensity and a lower proportion of positively stained cells in prostate cancer tissue. A similar trend in the expression of Cyclin D3, a partner protein of CDK11^p58^, was also observed.

As we reported before, CDK11^p58^ could inhibit the proliferation and promote the apoptosis of prostate cancer cells. To our surprise, as a Ser/Thr kinase, the proliferation inhibition was not fully kinase dependent. But the apoptosis promotion was dependent on it kinase activity.

As we speculated, CDK11^p58^ indeed inhibited the migration and invasion of AR + LNCap cells, but not AR- PC3 cell lines. These data demonstrated that CDK11^p58^ could inhibit the metastasis of prostate cancer cells through the AR signaling pathway. And also it was kinase dependent. Then we examined the interaction between CDK11^p58^ and AR. The data showed that T370A and T370D mutants were both capable of interacting with AR. The interaction was not totally dependent on its kinase activity. However, in our previous study, the other kinase dead mutant D224N failed to interact with AR. It suggested that the aspartic acid D224 of CDK11^p58^ was necessary for the interaction with AR not just because of its kinase activity. Maybe, the mutant D224N has changed the CDK11^p58^ protein configuration and finally influenced the interaction with AR. The exact mechanism needs further investigation. This result indicated both CDK11^p58^ and T370A could interact with AR, but only the kinase activated CDK11^p58^ could inhibit the metastasis of AR positive prostate cancer cells. Western Blot assay showed that metastasis-related genes integrin β3 and MMP2, but not MMP3 was inhibited by CDK11^p58^ in a dose and kinase dependent manner.

## Conclusions

Abnormal expression of CDK11^p58^ in prostate cancer tissue led to the dysfunction of cell apoptosis and metastasis of cancer. CDK11^p58^ inhibit the metastasis of AR positive prostate cancer cells through inhibition of integrin β3 and MMP2 in a kinase dependent manner. These data indicate that CDK11^p58^ is an anti-metastasis gene product in prostate cancer. Taken together, we demonstrate a new role for CDK11^p58^ as an anti-metastasis gene in prostate cancer.

## Methods

### Materials

Fetal bovine serum (FBS), Dulbecco modified Eagle medium (DMEM), 1640 and LipofectAMINE reagen were purchased from Invitrogen. The anti-cyclin D3 monoclonal antibody, the mouse and rabbit secondary antibody were purchased from Cell Signaling. The anti-CDK11 and anti-AR polyclonal antibody were purchased from Santa Cruz Biotechnology. HRP-conjugated goat anti-rabbit and HRP-conjugated goat anti-mouse IgG secondary antibodies were also purchased from Santa Cruz Biotechnology. The anti-MMP2 polyclonal antibody was purchased from Bioworld. The anti-MMP3 polyclonal antibody was purchased from Epitomics Company. The qRT-PCR assay system was purchased from Tiangen Company.

### Cell culture and cell transfections

The PC3 and LNCap cell lines were obtained from cell bank of our lab. Cells were grown using 1640 medium with 10% FBS, 100 units/ml penicillin, and 100 ug/ml streptomycin at 37°C and 5% CO_2_. Transfection for immunoprecipitation was performed in 100-mm dishes with 8 ug total plasmids.

### CDK11 Immunohistochemistry

Expression levels of CDK11 in postoperative paraffin-embedded tumor specimens from prostate cancer patients were detected with immunohistochemistry (IHC). The concentrations of antibody were as follows: anti-CDK11, 1:100 dilution; Nuclear expression of both CDK11 is defined as positive. The detailed staining procedures strictly followed the supplier’s recommendation. Negative controls were obtained by incubating parallel slides with the primary antibodies omitted. In addition, sections with confirmed positive staining from each run served as positive controls. Immunostaining of the whole slide area was evaluated by two independent pathologists who remained unaware of tumor characteristics and other staining results.

### Immunoprecipitation and western blotting

Prostate cancer cells were first lysed with ultrasoud in 1 ml of coimmunoprecipitation (CoIP) buffer (50 mM Tris–HCl (pH 7.5), 150 mM NaCl, 0.1% NP-40, 5 mM EDTA, 5 mM EGTA, 15 mM MgCl2, 60 mMβ-glycerophosphate, 0.1 mM sodium orthovanadate, 0.1 mM NaF, 0.1 mM benzamide, 10 *μ*g/ml aprotinin, 10 *μ*g/ml leupeptin, 1 mM PMSF). Detergent insoluble materials were removed by centrifugation. Whole tissue lysates were incubated with 2 *μ*g relevant antibody at 4°C for 2 h. Pre-equilibrated protein G-agarose beads were added and collected by centrifugation after incubation overnight and then gently washed three times with the lysis buffer. The bound proteins were eluted and analyzed using Western blots. An antibody to GAPDH was used to ensure equivalent loading.

### Transwell invasion

Cells invasion was assayed using BD biocoat growth factor reduced (GFR) matrigel invasion chambers (BD, CA). LNCap cell suspension (0.5 ml; 10×10^4^ cells/ml) was added to the inside of the inserts. Assays performed at 37°C, 5% CO2 were 24 h for transfected cells. After incubation, noninvading cells were removed from the upper surface of the membrane using cotton-tipped swabs. The cells on the lower surface of the membrane were stained with Crystal violet. Cells were counted in the central field of triplicate membranes.

### Confocal microscopy

LNCap cells grown to 50% confluence on coverslips were transiently transfected with indicated plasmids. After 48 h of transfection, cells were washed with PBS, fixed in 4% formaldehyde, permeabilized in 0.2% Triton X-100/PBS and blocked in 1% BSA for 1 h at room temperature. The coverslips were stained with anti-CDK11 and anti-AR antibody for 2 h at room temperature followed by incubation with fluorescein isothiocyanate (FITC)-conjugated goat anti rabbit secondary antibody and rhodamine-conjugated goat anti mouse for 1 h at room temperature. Cells were washed three times with PBS, stained for Hoechst 33258 (50 μg/ml) solution, respectively, in dark chamber. The coverslips were washed as described above, inverted, mounted on slides, and examined in a Zeiss or Leica TCS SP5 confocal microscope.

### Statistical analysis

The experimental data were expressed as the mean ± standard deviation, and the statistical significance between different groups was determined using t-tests. All statistical tests were two sided and P values less than 0.05 were considered significant.

## Competing interests

The authors declare that they have no competing interests.

## Authors’ contributions

XZ conceived and designed the study. YC performed the experiments. LW, XX analyzed the data. PW and YW contributed reagents, materials, and analysis tools. YC wrote the paper. All authors read and approved the final manuscript.
